# Differential Mueller matrix imaging of partially depolarizing optically anisotropic biological tissues

**DOI:** 10.1007/s10103-019-02878-2

**Published:** 2019-11-20

**Authors:** L. Trifonyuk, A. Sdobnov, W. Baranowski, V. Ushenko, O. Olar, A. Dubolazov, L. Pidkamin, M. Sidor, O. Vanchuliak, A. Motrich, M. Gorsky, I. Meglinski

**Affiliations:** 1 Rivne State Medical Center, 78 Kyivska Str, Rivne, 33007 Ukraine; 2 Faculty of Information Technology and Electrical Engineering, University of Oulu, 90570 Oulu, Finland; 3 Warsaw Military Institute of Medicine, 04141 Warsaw, Poland; 4 Chernivtsi National University, 2 Kotsiubynskyi Str, Chernivtsi, 58012 Ukraine; 5 Bukovinian State Medical University, 3 Theatral Sq, Chernivtsi, 58000 Ukraine; 6 Institute of Engineering Physics for Biomedicine (PhysBio), National Research Nuclear University MEPhI, Moscow, 115409 Russia; 7 Interdisciplinary Laboratory of Biophotonics, National Research Tomsk State University, Tomsk, 634050 Russia; 8 School of Engineering & Applied Science, Aston University, Birmingham, UK and School of Life & Health Sciences, Aston University, Aston University, Birmingham, UK

**Keywords:** Polarized light, Mueller matrix, Optical anisotropy, Birefringence, Partial depolarization, Biomedical imaging

## Abstract

Since recently, a number of innovative polarization-based optical imaging modalities have been introduced and extensively used in various biomedical applications, with an ultimate aim to attain the practical tool for the optical biopsy and functional characterization of biological tissues. The techniques utilize polarization properties of light and Mueller matrix mapping of microscopic images of histological sections of biological tissues or polycrystalline films of biological fluids. The main drawback of currently developed laser polarimetry approaches and Mueller matrix mapping techniques is poor reproducibility of experimental data. This is due to azimuthal dependence of polarization and ellipticity values of most matrix elements to sample orientation in respect to incidence light polarization. Current study aims to generalize the methods of laser polarimetry for diagnosis of partially depolarizing optically anisotropic biological tissues. A method of differential Mueller matrix mapping for reconstruction of linear and circular birefringence and dichroism parameter distributions of partially depolarizing layers of biological tissues of different morphological structure is introduced and practically implemented. The coordinate distributions of the value of the first-order differential matrix elements of histological sections of brain tissue with spatially structured, optically anisotropic fibrillar network, as well as of parenchymatous tissue of the rectum wall with an “islet” polycrystalline structure are determined. Within the statistical analysis of polarization reproduced distributions of the averaged parameters of phase and amplitude anisotropy, the significant sensitivity of the statistical moments of the third and fourth orders to changes in the polycrystalline structure of partially depolarizing layers of biological tissue is observed. The differentiation of female reproductive sphere connective tissue is realized with excellent accuracy. The differential Mueller matrix mapping method for reconstruction of distributions of linear and circular birefringence and dichroism parameters of partially depolarizing layers of biological tissues of different morphological structures is proposed and substantiated. Differential diagnostics of changes in the phase (good balanced accuracy) and amplitude (excellent balanced accuracy) of the anisotropy of the partially depolarizing layers of the vagina wall tissue with prolapse of the genitals is realized. The maximum diagnostic efficiency of the first-order differential matrix method was demonstrated in comparison with the traditional methods of polarization and Mueller matrix mapping of histological sections of light-scattering biological tissues.

## Introduction

The study of biological tissue structure in various pathological and physiological states is one of the most important tasks for modern microscopic biomedical imaging [1]. Morphologically, most biological tissues have anisotropic structure (fibrillar protein networks of eye tissues, skin derma,muscle, bone tissue, etc.) [2–4], which leads to the linear birefringence and dichroism. Additionally, many components (chiral molecules) of biological tissues have optical activity and circular dichroism [5–7]. Traditional light microscopy does not provide information on optical anisotropy, which limits its diagnostic capabilities. The main method for obtaining this information is the use of polarized radiation [8, 9]. Such techniques can be called a “polarization introscopy” (visualization of optical anisotropy parameters) of the polycrystalline structure of biological tissues. Particularly, Mueller matrix polarimetry (MMP) became one of the most effective polarization methods for tissue diagnostics [9]. This method provides the most complete information about the polarization manifestations of a set of mechanisms of optical anisotropy of biological objects [10, 11]. At the moment, MMP methods are developing in two main directions. The first one is the investigation of the structure and symmetry of the light scattering matrices (LSM)-angular dependences (indicatrices) of matrix elements [12, 13]. The second one is the investigation of Mueller matrix images (MMI)—coordinate distributions of matrix elements [14, 15]. Methods based on LSM allow obtaining statistically averaged information about the size, shape, and optical properties of ensembles of scattering structural elements of biological tissues [16–21]. The experimental determination of biological object MMI is an innovative development of LSM techniques [22]. The obtained MMI, as a superposition of depolarization, dichroism, and birefringence matrices, allow to investigate the integral structure of the optically anisotropic component of biological tissues [23, 24] using the polar decomposition method [25, 26]. This algorithm proved to be the most effective for analysis of the polycrystalline structure in the case of single scattering in biological layers. In this approximation, we first determined the Mueller matrix reconstruction algorithms for the coordinate distributions of the linear and circular birefringence and dichroism of biological tissues [27, 28]. Statistical analysis of the obtained maps of optical anisotropy revealed objective criteria (statistical moments of the first to fourth order), which were used as the basis for differentiation of oncological changes in human organs (benign and malignant tumors of the prostate, myometrium, and endometrium) [29–33]. The main limitation of this technique is the hardly achievable requirement of single scattering or the absence of depolarization. The most common type of laboratory samples of biological tissues is partially depolarizing optically anisotropic layers. Modern microscopic diagnosis of such changes does not provide a sufficient level of accuracy. For example, the accuracy of the analogous diagnosis of the first stage of endometrial cancer is 75%, the second stage is 66%, and only in the third stage it is increased to 88% [34]. At the preclinical stage of no less severe pathology of prolapse of the genitals, quantitative estimation of changes in the morphological structure of the connective tissue component of the uterine ligament is not precise enough [35, 36]. Therefore, the further development of the MMP method in the diagnostics of the polycrystalline structure of light-scattering biological tissues of various morphological structures and the physiological state is relevant. One of the ways to solve this problem is described in references [37–43], which are based on their presentation of the Mueller matrix as a superposition of a completely polarized (first-order differential matrix) and depolarized (second-order differential matrix) components. Differential, in contrast to traditional [14–22] Mueller matrix mapping and polar decomposition methods [25, 26], provides separate information about the distribution of average values (polarized component of the Mueller matrix) and magnitudes (depolarized component of the Mueller matrix) of fluctuations of the phase and amplitude anisotropy parameters.

However, this theoretical differential Mueller matrix method has not yet found practical application in biomedical diagnostics.

Current paper is aimed to the development and experimental approbation of the method of differential Mueller matrix mapping and reconstruction of the parameters of optical anisotropy of the partially depolarizing layers of biological tissues of various morphological structures (brain, rectal wall) and the pathological state “norm-prolapse of genitals” in order to obtain quantitative criteria for evaluating and differentiating the polycrystalline structure of such objects.

## Theoretical background

In this section, we give a brief outline of the theory [37–43] which describes algorithms for experimental measurement of a first-order differential matrix of a partially depolarizing optically anisotropic biological layer. In the case of multiple scattering, the Mueller matrix of a depolarizing layer varies along the propagation direction of light z. Analytically, this dependence is illustrated by the equation

1 $$ \frac{\mathrm{d}\ \left\{\mathrm{M}\ \right\}\left(\mathrm{z}\right)}{\mathrm{d}\mathrm{z}}-\left\{M\right\}\left\{z\right\}\left\{m\right\}(z) $$

where {*M*}(*z*) is the Mueller matrix object in the plane z and {*m*}(*z*) is the differential Mueller matrix.

For optically thin layers, the non-depolarizing differential matrix {*m*}(*z*) consists of six elementary polarization properties which fully characterize the optical anisotropy of a biological layer. These parameters fully characterize the amplitude (linear *LD*_0; 90_, *LD*_45; 135_, and circular *CD*_⊗; ⊕_ dichroism) and phase (linear *LB*_0; 90_, *LB*_45; 135_, and circular *CB*_⊗; ⊕_ birefringence) anisotropy of the biological layer

2 $$ {\displaystyle \begin{array}{l}\left\{m\right\}=\left\Vert \begin{array}{ccc}0& {m}_{12}& {m}_{13}\kern0.5em {m}_{14}\\ {}{m}_{21}& 0& {m}_{23}\kern0.5em -{m}_{24}\\ {}\begin{array}{c}{m}_{31}\\ {}{m}_{41}\end{array}& \begin{array}{c}-{m}_{32}\\ {}{m}_{42}\end{array}& \begin{array}{c}\begin{array}{cc}0& {m}_{34}\end{array}\\ {}\begin{array}{cc}-{m}_{43}& 0\end{array}\end{array}\end{array}\right\Vert \\ {}=\left\Vert \begin{array}{ccc}0& {LD}_{0;90}& \begin{array}{cc}{LD}_{45;135}& {CD}_{\otimes; \oplus}\end{array}\\ {}{LD}_{0;90}& 0& \begin{array}{cc}{CB}_{\otimes; \oplus }& -{LB}_{45;135}\end{array}\\ {}\begin{array}{c}{LD}_{45;135}\\ {}{CD}_{\otimes; \oplus}\end{array}& \begin{array}{c}-{CB}_{\otimes; \oplus}\\ {}{LB}_{45;135}\end{array}& \begin{array}{c}\begin{array}{cc}0& {LB}_{0;90}\end{array}\\ {}\begin{array}{cc}-{LB}_{0;90}& 0\end{array}\end{array}\end{array}\right\Vert \end{array}} $$

Here, the indices “0; 90”, “45; 135” denote the unit vectors of the linearly polarized components; ⊗ right, ⊕ left circularly polarized components of the amplitude of the laser wave.

The partially depolarizing medium for expression (2) can be represented as average 〈{*m*}〉 (polarization part {*m*}(*z*)) and fluctuating〈{*Δm*^2^}〉 (depolarizing part {*m*}(*z*)) components

3 $$ \left\{m\right\}(z)=\left\langle \left\{m\right\}\right\rangle +\left\langle \left\{\varDelta {m}^2\right\}\right\rangle $$

It should be noted that there is always a feedback between the differential matrix and the Mueller matrix

4 $$ M(z)=\exp \left(\left\{m\right\}(z)\right) $$

A combined analysis of ratios (1)-(4) is performed and a logarithmic expression of matrix algorithm is derived as

5 $$ L(z)=\ln \left\{M(z)\right\}={L}_p+{L}_d $$

which is determined as superposition of antisymmetric *Lp* (polarization) and symmetric *Ld* (depolarization) components of *L*(*z*)

6 $$ \left\{\begin{array}{l}{L}_p=\left\langle \left\{m\right\}\right\rangle z;\\ {}{L}_d=0,5\left\langle \left\{\varDelta {m}^2\right\}\right\rangle {z}^2,\end{array}\right. $$

where

7 $$ \left\{\begin{array}{l}{L}_p=0.5\left(L-G{L}^TG\right);\\ {}{L}_d=0.5\left(L+G{L}^TG\right);\\ {}G=\mathit{\operatorname{diag}}\left(1,-1,-1,-1\right).\end{array}\right. $$

Here, *T* is the transpose operation and *G* is the Minkowski metric matrix.

Taking into account relations (2)–(7), polarization component of a logarithmic matrix algorithm L(z) takes the form of

8 $$ {L}_p=\left\Vert \begin{array}{ccc}0& \left({j}_{12}+{j}_{21}\right)& \left({j}_{13}+{l}_{31}\right)\kern0.5em \left({j}_{14}+{j}_{41}\right)\\ {}\left({j}_{21}+{l}_{12}\right)& 0& \begin{array}{cc}\left({j}_{23}-{j}_{32}\right)& \left({j}_{24}-{j}_{42}\right)\end{array}\\ {}\begin{array}{c}\left({j}_{31}+{l}_{13}\right)\\ {}\left({j}_{41}+{l}_{14}\right)\end{array}& \begin{array}{c}\left({j}_{32}-{l}_{23}\right)\\ {}\left({j}_{42}-{l}_{24}\right)\end{array}& \begin{array}{c}\begin{array}{cc}0& \left({j}_{34}-{j}_{43}\right)\end{array}\\ {}\begin{array}{cc}\left({j}_{43}-{j}_{34}\right)& 0\end{array}\end{array}\end{array}\right\Vert $$

where

9 $$ \left\{\begin{array}{l}{j}_{ik}=\ln {M}_{ik};\\ {}{j}_{ik}+{j}_{ki}=\ln \left({M}_{ik}\times {M}_{ki}\right);\\ {}{j}_{ik}-{j}_{ki}=\ln \left(\frac{M_{ik}}{M_{ki}}\right)\end{array}\right. $$

On the basis of relations (8) and (9), we found a relationship between the elements of the first-order differential matrix〈{*m*_*ik*_}〉 (equations (2) and (3)) and combinations of the averaged by depth l elements *M*_*ik*_of the Mueller matrix (equation (3)) of the partially depolarizing layer of the biological fabrics

10 $$ \left\langle \left\{m\right\}\right\rangle ={\boldsymbol{z}}^{-1}\times {L}_p={\boldsymbol{z}}^{-1}\times \left\Vert \begin{array}{ccc}0& \ln \left({M}_{12}{M}_{21}\right)& \ln \left({M}_{13}{M}_{31}\right)\kern0.5em \ln \left({M}_{14}{M}_{41}\right)\\ {}\ln \left({M}_{12}{M}_{21}\right)& 0& \begin{array}{cc}\ln \left(\frac{M_{23}}{M_{32}}\right)& \ln \left(\frac{M_{24}}{M_{42}}\right)\end{array}\\ {}\begin{array}{c}\ln \left({M}_{13}{M}_{31}\right)\\ {}\ln \left({M}_{14}{M}_{41}\right)\end{array}& \begin{array}{c}\ln \left(\frac{M_{32}}{M_{23}}\right)\\ {}\ln \left(\frac{M_{42}}{M_{24}}\right)\end{array}& \begin{array}{cc}\begin{array}{c}0\\ {}\ln \left(\frac{M_{43}}{M_{34}}\right)\end{array}& \begin{array}{c}\ln \left(\frac{M_{34}}{M_{43}}\right)\\ {}0\end{array}\end{array}\end{array}\right\Vert $$

The combined analysis of relations (2) and (10) allowed us to obtain algorithms of polarization reconstruction of average values (over the entire depth l of the biological layer) of parameters of phase and amplitude anisotropy of polycrystalline structure of optically thick biological layer

11 $$ L{B}_{0;90}={l}^{-1}\ln \left(\frac{M_{34}}{M_{43}}\right) $$

12 $$ L{B}_{45;135}={l}^{-1}\ln \left(\frac{M_{24}}{M_{42}}\right) $$

13 $$ C{B}_{\otimes; \oplus }={l}^{-1}\ln \left(\frac{M_{23}}{M_{32}}\right) $$

14 $$ {LD}_{0;90}={l}^{-1}\mathit{\ln}\left({M}_{12}{M}_{21}\right) $$

15 $$ {\mathrm{LD}}_{45;135}={l}^{-1}\ln \left({M}_{13}{M}_{31}\right) $$

16 $$ C{D}_{\otimes; \oplus }={l}^{-1}\ln \left({M}_{14}{M}_{41}\right) $$

Thus, the use of differential analysis of Mueller matrix mapping data allowed us to obtain a set of algorithms (ratios (11)–(16)) of polarization reconstruction of average values of phase and amplitude anisotropy parameters of polycrystalline component of partially depolarized biological layer.

### Experimental approach

In this part of the paper, the theory for experimental mapping of the distributions of elements of a first-order differential matrix 〈{*m*_*ik*_}〉using the MMP technique is presented. As a lighting probe, linearly polarized (with azimuths 0°, 45°, 90°) and right circularly polarized helium-neon laser beams (wavelength 632.8 *μm*, power 10 mW) were used in series.

For each of the illuminating beams, a polarization analysis of the microscopic image of the biological layer was carried out. For this purpose, multichannel polarization filtering was used. More detailed description can be found in our previous papers [27–29]. Therefore, we do not give a description of the computation of the elements *M*_*ik*_, but the main attention is paid to the methods for determination of the distributions〈{*m*_*ik*_}〉.

Taking into account relations (8)–(10), we obtain the expressions for calculation of elements of differential matrix of the first order, which characterize the linear and circular:

- birefringence ($$ L{B}_{0,90};\kern0.5em L{B}_{45,135};\kern0.5em C{B}_{\otimes, \oplus } $$):

17 $$ \left.\begin{array}{l}\left\langle {m}_{34}\right\rangle =\ln \left(\frac{M_{34}}{M_{43}}\right)=\ln \left(\frac{S_3^{\otimes }-0,5\left({S}_3^0+{S}_3^{90}\right)}{S_4^{45}-0,5\left({S}_4^0+{S}_4^{90}\right)}\right)\\ {}\left\langle {m}_{43}\right\rangle =\ln \left(\frac{M_{43}}{M_{34}}\right)=\ln \left(\frac{S_4^{45}-0,5\left({S}_4^0+{S}_4^{90}\right)}{S_3^{\otimes }-0,5\left({S}_3^0+{S}_3^{90}\right)}\right)\end{array}\right\}\Rightarrow L{B}_{0,90}; $$

18 $$ \left.\begin{array}{l}\left\langle {m}_{42}\right\rangle =\ln \left(\frac{M_{42}}{M_{24}}\right)=\ln \left(\frac{0,5\left({S}_4^0-{S}_4^{90}\right)}{S_2^{\otimes }-0,5\left({S}_2^0+{S}_2^{90}\right)}\right)\\ {}\left\langle {m}_{24}\right\rangle =\ln \left(\frac{M_{24}}{M_{42}}\right)=\ln \left(\frac{S_2^{\otimes }-0,5\left({S}_2^0+{S}_2^{90}\right)}{0,5\left({S}_4^0-{S}_4^{90}\right)}\right)\end{array}\right\}\Rightarrow L{B}_{45,135}; $$

19 $$ \left.\begin{array}{l}\left\langle {m}_{23}\right\rangle =\ln \left(\frac{M_{23}}{M_{32}}\right)=\ln \left(\frac{S_2^{45}-0,5\left({S}_2^0+{S}_2^{90}\right)}{0,5\left({S}_3^0-{S}_3^{90}\right)}\right)\\ {}\left\langle {m}_{32}\right\rangle =\ln \left(\frac{M_{32}}{M_{23}}\right)=\ln \left(\frac{0,5\left({S}_3^0-{S}_3^{90}\right)}{S_2^{45}-0,5\left({S}_2^0+{S}_2^{90}\right)}\right)\end{array}\right\}\Rightarrow C{B}_{\otimes, \oplus }; $$

- dichroism ($$ L{D}_{0,90};\kern0.5em L{D}_{45,135};\kern0.5em C{D}_{\otimes, \oplus } $$):

20 $$ {\displaystyle \begin{array}{l}\left\langle {m}_{12}\right\rangle =\left\langle {m}_{21}\right\rangle =\ln \left({M}_{12}{M}_{21}\right)=\\ {}=\ln \left(0,25\left({S}_1^0-{S}_1^{90}\right)\left({S}_2^0+{S}_2^{90}\right)\right)\Rightarrow L{D}_{0,90}\end{array}}; $$

21 $$ {\displaystyle \begin{array}{l}\left\langle {m}_{13}\right\rangle =\left\langle {m}_{31}\right\rangle =\ln \left({M}_{13}{M}_{31}\right)=\\ {}=\ln \left(0,25\left({S}_1^{45}-0,5\left({S}_1^0+{S}_1^{90}\right)\right)\left({S}_3^0+{S}_3^{90}\right)\right)\Rightarrow L{D}_{45,135}\end{array}}; $$

22 $$ {\displaystyle \begin{array}{l}\left\langle {m}_{14}\right\rangle =\left\langle {m}_{41}\right\rangle =\ln \left({M}_{14}{M}_{41}\right)=\\ {}=\ln \left(0,25\left({S}_1^{\otimes }-0,5\left({S}_1^0+{S}_1^{90}\right)\right)\left({S}_4^0+{S}_4^{90}\right)\right)\Rightarrow C{D}_{\otimes, \oplus}\end{array}}. $$

23 $$ {\displaystyle \begin{array}{c}{S}_{i=1}^{0;45;90;\otimes }={I}_0^{0;45;90;\otimes }+{I}_{90}^{0;45;90;\otimes };\\ {}{S}_{i=2}^{0;45;90;\otimes }={I}_0^{0;45;90;\otimes }-{I}_{90}^{0;45;90;\otimes };\\ {}\begin{array}{c}{S}_{i=3}^{0;45;90;\otimes }={I}_{45}^{0;45;90;\otimes }-{I}_{135}^{0;45;90;\otimes };\\ {}{S}_{i=4}^{0;45;90;\otimes }={I}_{\otimes}^{0;45;90;\otimes }+{I}_{\oplus}^{0;45;90;\otimes }.\end{array}\end{array}} $$

Here, ${S}_{i=1;2;3;4}^{0;45;90;\otimes }$is the parameters of the Stokes vector of an image of a partially depolarizing histological section of biological tissue; *I*_0; 45; 90; 135; ⊗ ; ⊕_ is the intensity of light transmitted through the object that passed through the linear polarizer with the rotation angle of the transmission plane *Θ*:${0}^0;\kern0.5em {45}^0;\kern0.5em {90}^0;\kern0.5em {135}^0$, and through a system of “quarter wave plate polarizer” of polarization analysis unit that transmits right (⊗) and left (⊕) circularly polarized components of the object laser radiation [24]. Within the ensemble (*m* × *n*) of CCD camera pixels (The Imaging Source DMK41AU02.AS, monochrome 1/2″ CCD, Sony ICX205AL (progressive scan); resolution—1280 × 960; size of light-sensitive plate—5952 m × 4464 m; sensitivity—0.05 lx; dynamic range—8 bit; SNR—9 bit, nonlinearity does not exceed 3–5%), we obtain the coordinate distributions of the elements of the first-order differential matrix.

The next step is the statistical analysis of the optical anisotropy maps obtained with the help of algorithms (17)–(23) $$ \left(\begin{array}{l}L{B}_{0,90};L{B}_{45;135};C{B}_{\otimes, \oplus };\\ {}L{D}_{0,90};L{D}_{45;135};C{D}_{\otimes, \oplus}\end{array}\right)\left(m\times n\right) $$the calculation of the statistical moments of the first to fourth orders *Z*_*i* = 1; 2; 3; 4_which characterize the average (*Z*_1_), dispersion (*Z*_2_), skewness (*Z*_3_), and kurtosis (*Z*_4_) of the coordinate distributions of the values of the phase and amplitude anisotropy of the polycrystalline structure of a sample of biological tissue [29].

### Brief description of the facilities

Our choice of the objects of study was based on the following fundamental and applied considerations.

*Fundamental aspect*—there are two limiting cases in the structure of optically anisotropic biological layers [1, 9, 14, 21]. The first case—spatially structured fibrillary networks. For such networks, due to availability of the “far” order, the mechanism of linear birefringence prevails on the background of circular birefringence of chiral molecules $$ {\displaystyle \begin{array}{l}L{B}_{0,90};L{B}_{45;135}>C{B}_{\otimes, \oplus };\\ {}L{D}_{0,90};L{D}_{45;135}>C{D}_{\otimes, \oplus}\end{array}} $$. As an example of such a tissue, we chose a histological section of the brain tissue of dead patients. The polycrystalline structure of this object is mainly formed by large-scale (up to 10 cm) fibrillary nerve fiber networks (*LB*_0; 90_; *LB*_45; 135_; *LD*_0; 90_; *LD*_45; 135_). In addition, the neuron networks of the brain tissue contain thin filamentary neurofibrils, which are formed by neuroalbumins (*LB*_0; 90_; *LB*_45; 135_; *LD*_0; 90_; *LD*_45; 135_) and neuroglobulins (*CB*_⊗; ⊕_; *CD*_⊗; ⊕_).

The second one—parenchymatous or “islet” optically anisotropic structures. For such formations, there is no “far” due to a threadlike fibrous network. The predominant type of morphological structure here is the presence of spatially non-oriented polypeptide chains that are generated by optically active molecules with circular birefringence. In other words, $$ {\displaystyle \begin{array}{l}L{B}_{0,90};L{B}_{45;135}\le C{B}_{\otimes, \oplus };\\ {}L{D}_{0,90};L{D}_{45;135}\le C{D}_{\otimes, \oplus}\end{array}} $$. As an example of such tissue, we chose histological section of the rectum wall. The morphological structure of this tissue is formed by three layers. The inner layer is mucous on the basis of epithelial tissue (*CB*_⊗, ⊕_; *CD*_⊗, ⊕_). Muscular layer formed by smooth myocytes ($$ {\displaystyle \begin{array}{l}L{B}_{0,90};L{B}_{45;135}\approx C{B}_{\otimes, \oplus };\\ {}L{D}_{0,90};L{D}_{45;135}\approx C{D}_{\otimes, \oplus}\end{array}} $$) and fibers of loose connective tissue ($$ {\displaystyle \begin{array}{l}L{B}_{0,90};L{B}_{45;135};\\ {}L{D}_{0,90};L{D}_{45;135}\end{array}} $$).

The study of differential matrices of the first order of selected samples provides information on the magnitude and ranges of the variation of the statistical parameters (*Z*_*i* = 1; 2; 3; 4_), which characterize the distribution of phase (*LB*_0, 90_; *LB*_45; 135_; *CB*_⊗, ⊕_) and amplitude (*LD*_0, 90_; *LD*_45; 135_; *CD*_⊗, ⊕_) anisotropy of biological tissues with different polycrystalline structure.

*Applied aspect*—the actual task of differential Mueller matrix mapping is the determination of the criteria for differentiation between healthy and pathologically altered samples of the human body’s biological tissue. As an example, we chose the histological sections of the operatively extracted vaginal wall during the prolapse of genitals of two types—healthy (group 1) and pathologically altered (group 2) patients. We already mentioned that at the preclinical stage of no less severe pathology, quantitative estimation of changes in the morphological structure of the connective tissue component of the uterine ligament is ineffective [35, 36]. The morphological structure of the vaginal wall is formed by the mucosa with the epithelial base (*CB*_⊗, ⊕_; *CD*_⊗, ⊕_) and the fibers of the loose connective tissue ($$ {\displaystyle \begin{array}{l}L{B}_{0,90};L{B}_{45;135};\\ {}L{D}_{0,90};L{D}_{45;135}\end{array}} $$), as well as the muscular fibrillar spiral and longitudinal layers of the myosin fibrils ($$ {\displaystyle \begin{array}{l}L{B}_{0,90};L{B}_{45;135};\\ {}L{D}_{0,90};L{D}_{45;135}\end{array}} $$). Prolapse of the genitals is accompanied by destructuring and thinning of the fibrillar nets of the muscular layer. This pathological process leads to a decrease in structural optical anisotropy. Therefore, the obtained optical anisotropy maps $$ \left(\begin{array}{l}L{B}_{0,90};L{B}_{45;135};C{B}_{\otimes, \oplus };\\ {}L{D}_{0,90};L{D}_{45;135};C{D}_{\otimes, \oplus}\end{array}\right)\left(m\times n\right) $$can be relevant for the development of criteria for early (preclinical) diagnosis of this pathology at a stage when there are no obvious morphological changes in the vaginal wall.

Histological sections of all types of biological tissues were made according to the standard method on a microtome with freezing. The obtained samples are characterized by the following optical geometric parameters:

- brain tissue-geometric thickness *l* = 60*μm*; coefficient of attenuation (extinction) *τ* = 0.21; degree of depolarization of laser radiation *Λ* = 43%;
- rectal wall tissue-*l* = 60*μm*; *τ* = 0.32; *Λ* = 58%;
- tissue samples of the vaginal wall-*l* = 60*μm*; *τ* = 0.26 ÷ 0.29; *Λ* = 47 % − 52%.

**Fig. 1:**
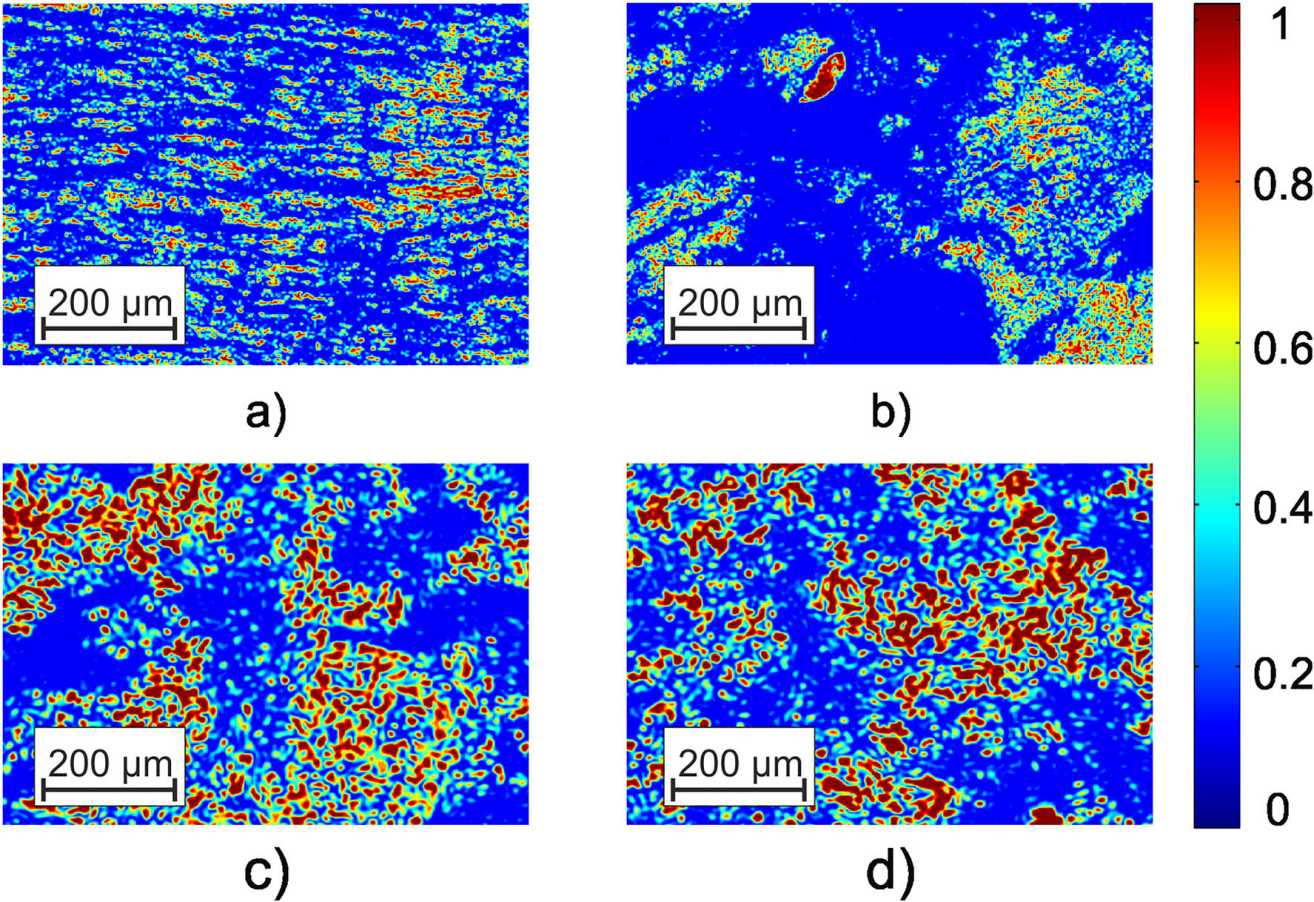
Polarizationally visualized images of an optically anisotropic structure of histological sections of brain (fragment (**a**), *l* = 60*μm*; *τ* = 0.21; *Λ* = 43%), of rectal wall (fragment (**b**), *l* = 60*μm*; *τ* = 0.32; *Λ* = 58%) tissues, healthy (fragment (**c**), *l* = 60*μm*; *τ* = 0.27; *Λ* = 49%), and pathologically altered (fragment (**d**), *l* = 60*μm*; *τ* = 0.29; *Λ* = 52%) vagina wall. See explanation in the text

Figure 1 shows microscopic images (×4) of the polycrystalline structure of samples of biological layers, polarizationally visualized in crossed polarizer-analyzer, namely, partially depolarizing histological sections of brain (fragment (1)), rectal wall (fragment (2)), healthy (fragment (3)), and pathologically altered (fragment (4)) vagina wall tissues.

The polarization images of biological tissue histological sections presented in Fig. 1 illustrate the diversity of topographical and geometric structure (bright areas) of optically anisotropic structures:

- birefringent spatially structured fibrillar nerve fiber networks of brain tissue (fragment (a));
- “islands” of optically active birefringent fibers of loose connective tissue of the rectum wall (fragment (b));
- optically anisotropic fibers of loose connective tissue and
- ibrillar myosin nets of healthy (fragment (c)) and pathologically altered (fragment (d)) vaginal wall.

As can be seen (fragments (a) and (b)), the topographic structure of the polarization images of optically anisotropic tissue of different morphological structures is significantly different both in size and shape. In contrast, a comparative qualitative analysis of images of histological sections of the vaginal wall samples revealed a similarity not only of morphological, but also polycrystalline structure.

## Results and discussion

**Fig. 2:**
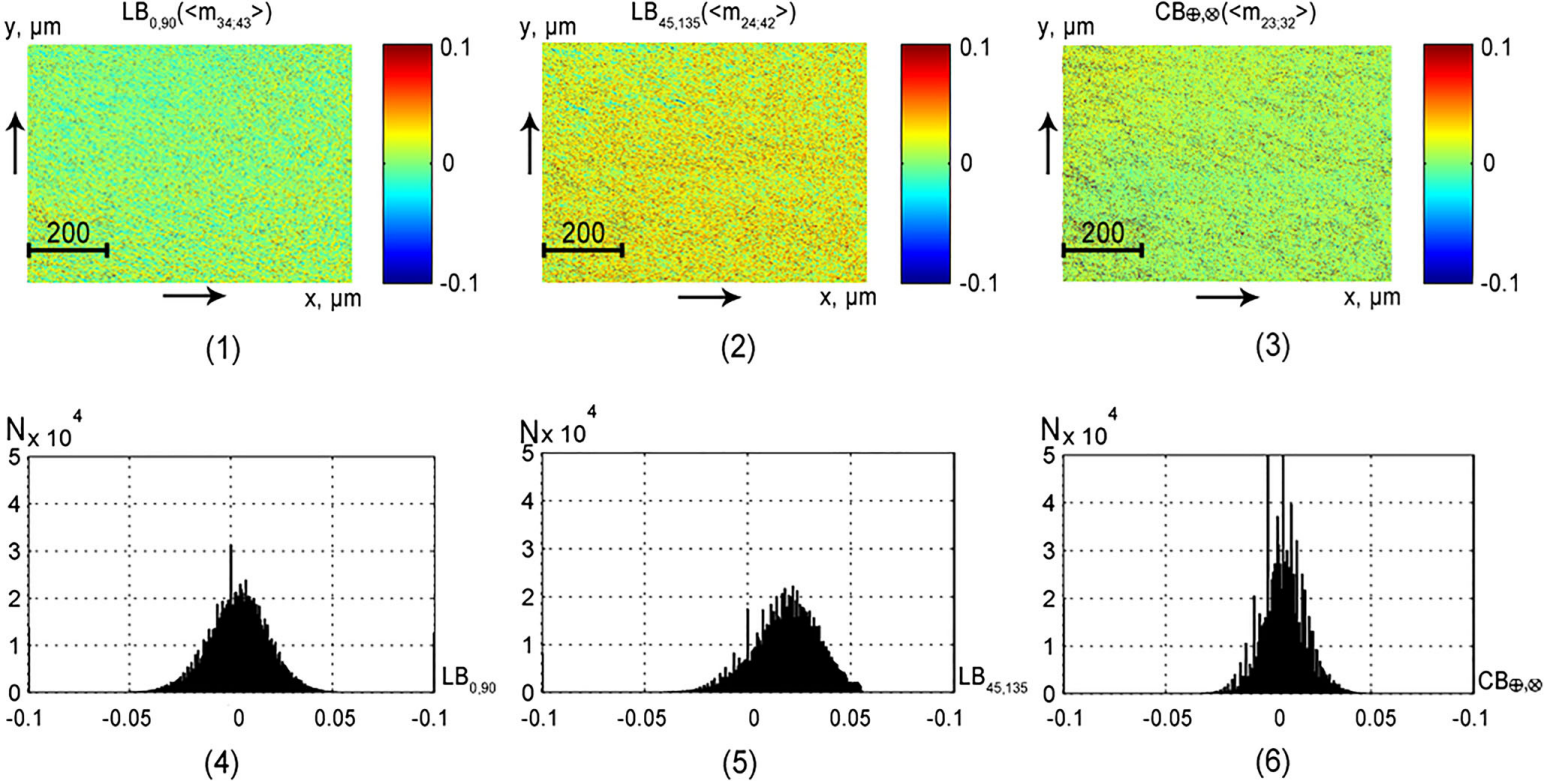
Maps (1–3) and histograms (4–6) of the partially depolarizing layer of brain tissue phase anisotropy parameters (*l* = 60*μm*; *τ* = 0.21; *Λ* = 43%)

**Fig. 3:**
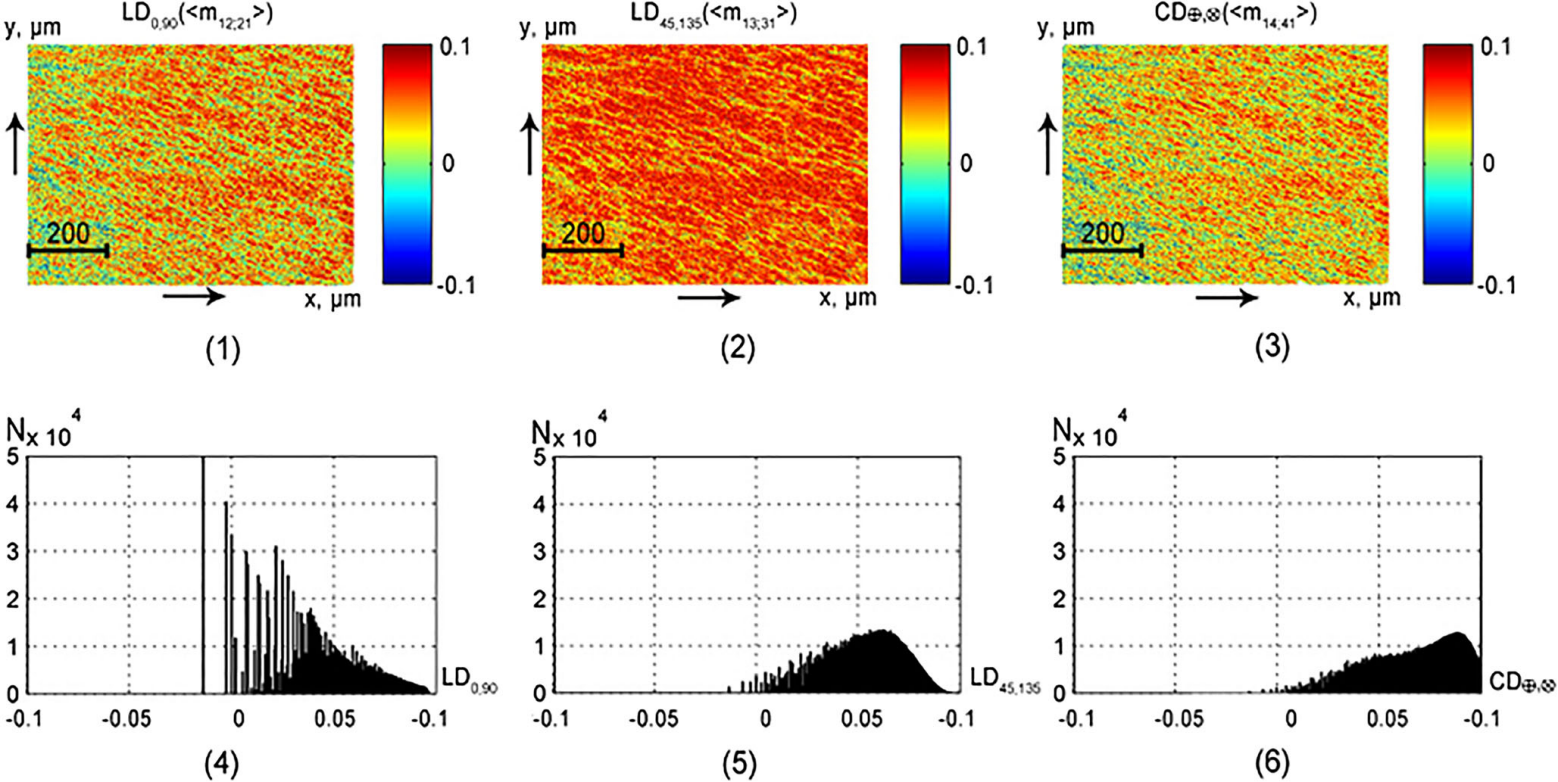
Maps (1–3) and histograms (4–6) of the partially depolarizing layer of brain tissue amplitude anisotropy parameters (*l* = 60*μm*; *τ* = 0.21; *Λ* = 43%)

**Fig. 4:**
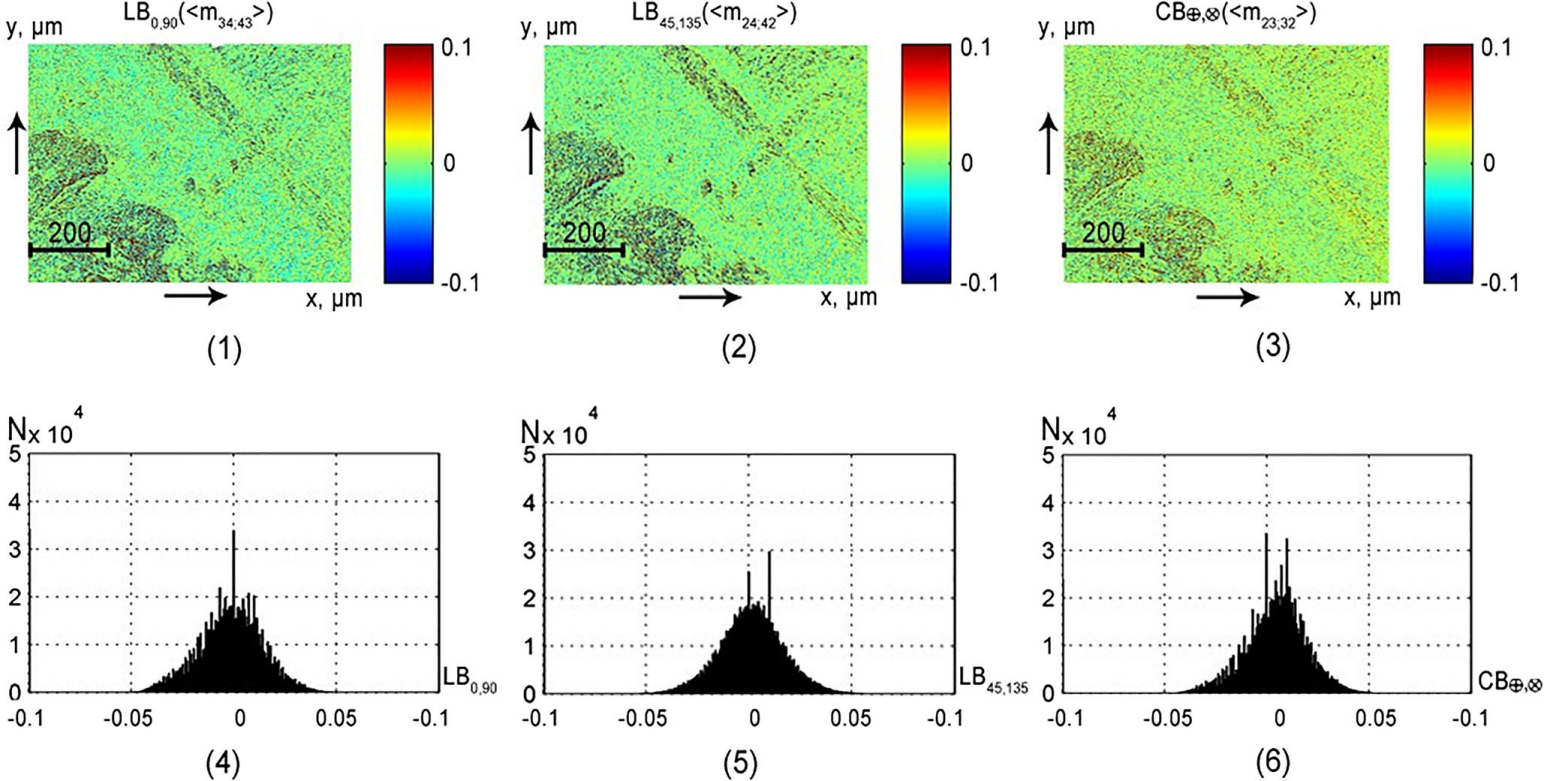
Maps (1–3) and histograms (4–6) of the partially depolarizing layer of rectum wall tissue phase anisotropy parameters (*l* = 60*μm*; *τ* = 0.32; *Λ* = 58%)

**Fig. 5:**
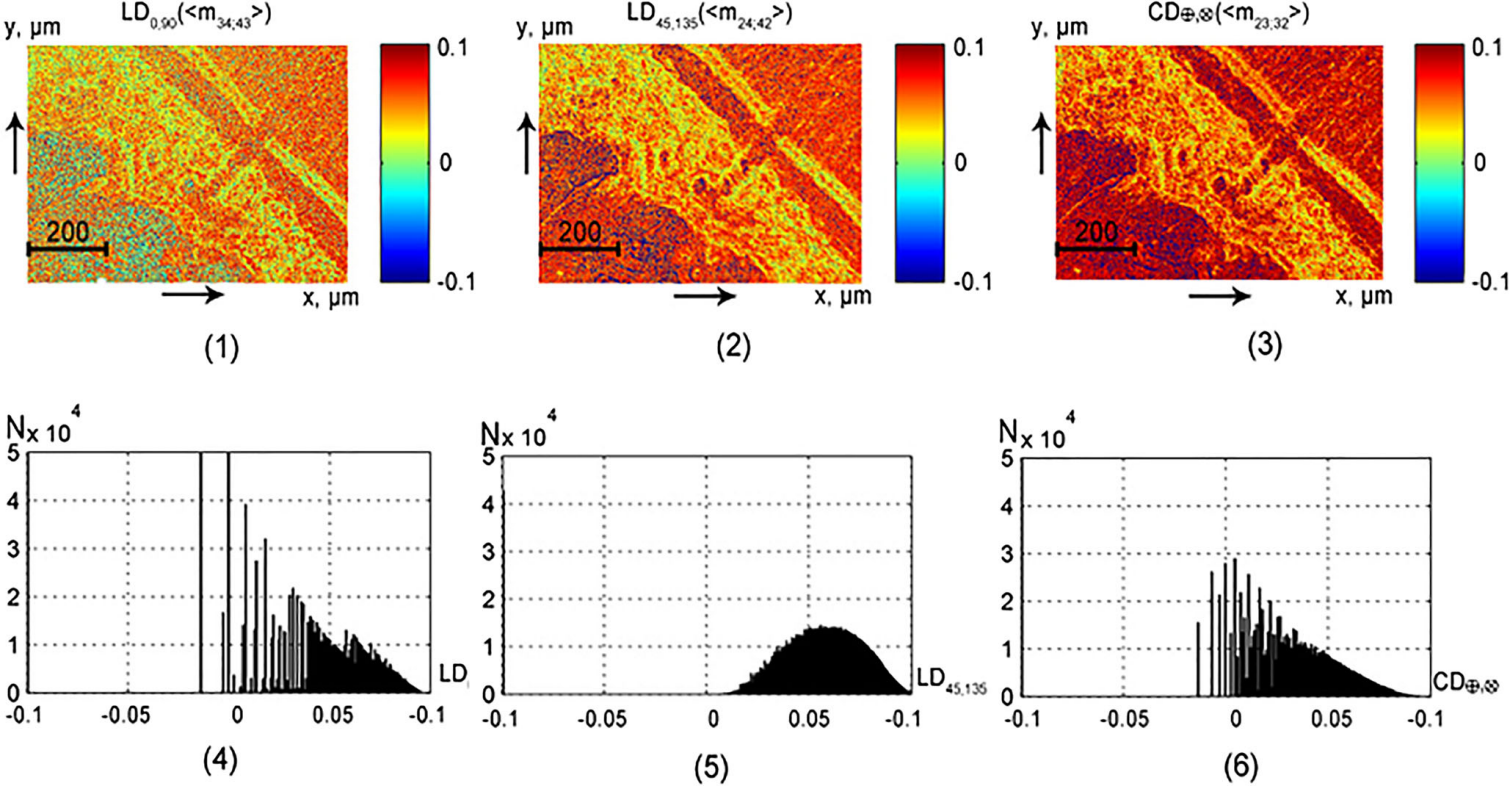
Maps (1–3) and histograms (4–6) of the partially depolarizing layer of rectum wall tissue amplitude anisotropy parameters (*l* = 60*μm*; *τ* = 0.32; *Λ* = 58%)

**Fig. 6:**
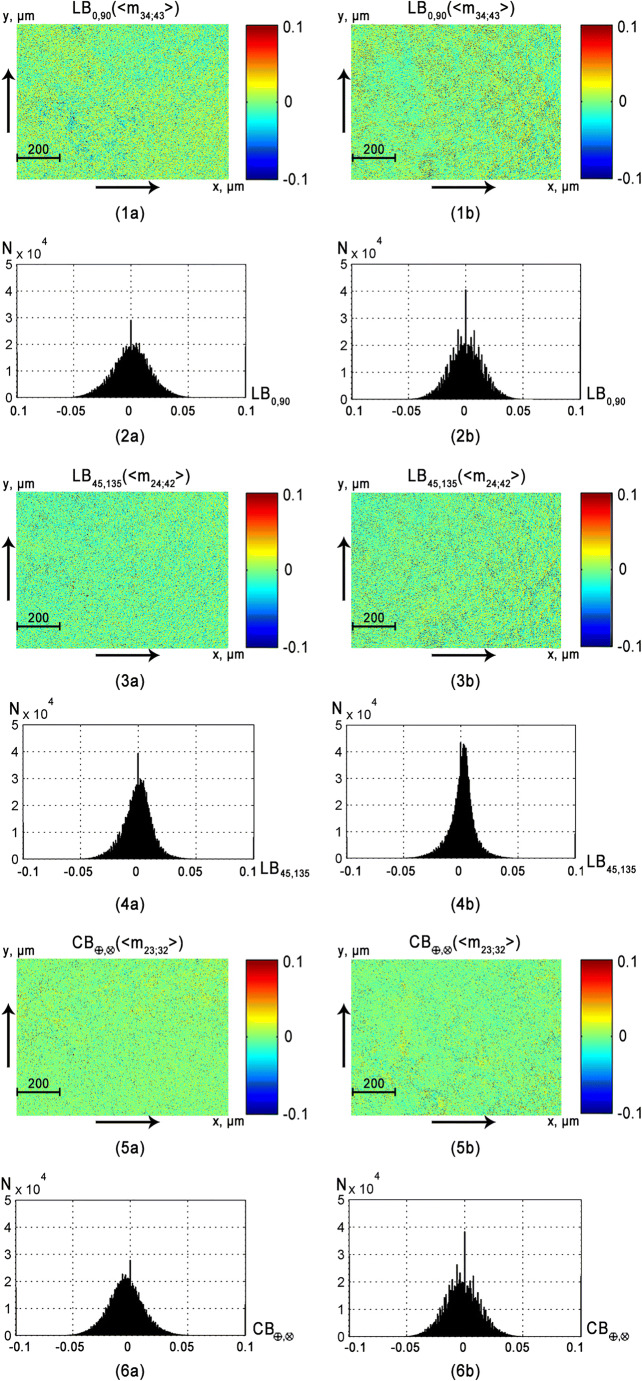
Maps (**1a**, **1b**, **3a**, **3b**) and histograms (**2a**, **2b**, **4a**, **4b**) of the distributions of the vaginal wall samples from group 1 (fragments **1a**–**6a**) and group 2 (fragments **1b**–**6b**) phase (*LB*_0; 90_; *LB*_45; 135_; *CB*_⊗; ⊕_) anisotropy

**Fig. 7:**
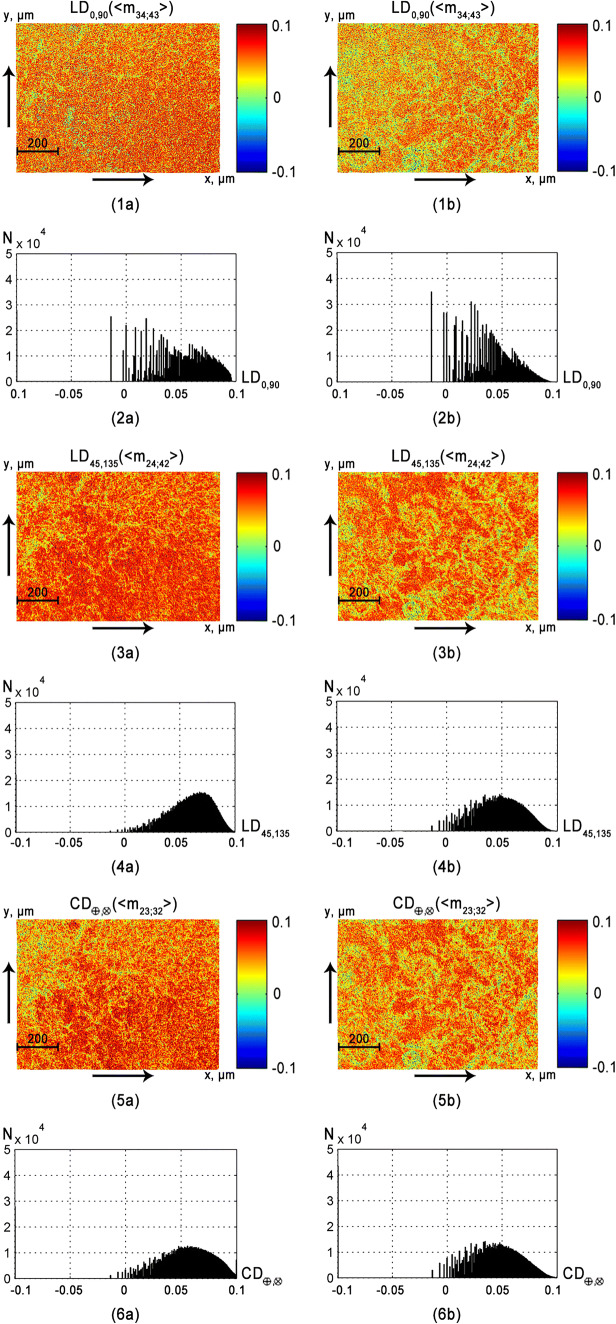
Maps (**1a**, **1b**, **3a**, **3b**) and histograms (**2a**, **2b**, **4a**, **4b**) of the distributions of the vaginal wall samples from group 1 (fragments **1a**–**6a**) and group 2 (fragments **1b**–**6b**) amplitude (*LB*_0; 90_; *LB*_45; 135_; *CB*_⊗; ⊕_) anisotropy

This part of the article presents the results of experimental approbation of the method of differential Mueller matrix mapping of histological sections of partially depolarizing biological tissues of different morphological structure and physiological state with the aim of:

- studying the structure and symmetry of first-order differential matrices of all types of samples (Figs. 2, 3, 4, and 5);
- determination of the magnitude and ranges of the changes in the statistical moments of the first to fourth orders *Z*_*i* = 1; 2; 3; 4_, which characterize the distributions$$ {\displaystyle \begin{array}{l}L{B}_{0,90};L{B}_{45;135};C{B}_{\otimes, \oplus };\\ {}L{D}_{0,90};L{D}_{45;135};C{D}_{\otimes, \oplus}\end{array}} $$(Tables 1 and 2);
- finding objective criteria (*Z*_*i* = 1; 2; 3; 4_) for differentiating the polycrystalline structure of healthy and pathologically altered tissues of the vaginal wall during genital prolapse (Figs. 6 and 7, Tables 3 and 4);
- comparative analysis of the diagnostic efficiency of our method and the traditional methods of direct polarization and Mueller matrix mapping (Table 5).

**Table 1:** Statistical moments of the first to fourth orders characterizing distributions of value of optical anisotropy parameters of a histological section of brain tissue (*z* = 60*μm*; *τ* = 0, 21; *Λ* = 43%)

*Z*_*i*_	*LB*_0; 90_	*LB*_45; 135_	*CB*_⊗; ⊕_	*LD*_0; 90_	*LD*_45; 135_	*CD*_⊗; ⊕_
*Z*_1_	0.015	0.025	0.01	0.074	0.085	0.065
*Z*_2_	0.03	0.04	0.02	0.087	0.07	0.06
*Z*_3_	0.11*	0.21	0.17*	0.82*	0.57	1.37*
*Z*_4_	0.18*	0.27	0.23*	1.12*	0.33	1.76*

**Table 2:** Statistical moments of the first to fourth orders, which characterize the distribution of the of optical anisotropy parameters of rectum wall (*l* = 60*μm*; *τ* = 0.32; *Λ* = 58%) histological section

*Z*_*i*_	*LB*_0; 90_	*LB*_45; 135_	*CB*_⊗; ⊕_	*LD*_0; 90_	*LD*_45; 135_	*CD*_⊗; ⊕_
*Z*_1_	0.011	0.017	0.015	0.055	0.05	0.025
*Z*_2_	0.02	0.025	0.02	0.048	0.06	0.04
*Z*_3_	0.38*	0.24*	0.41	0.52*	0.18*	0.97
*Z*_4_	0.44*	0.32*	0.53	0.88*	0.34*	0.58

**Table 3:** Statistical moments of the first to fourth orders characterizing distributions (*LB*_0, 90_; *LB*_45; 135_; *CB*_⊗, ⊕_) of vagina wall histological sections of the patients from group 1 and group 2, and balanced accuracy of the method

*Z*_*i*_	*LB*_0, 90_		*Ac*, %	*LB*_45; 135_		*Ac*, %	*CB*_⊗, ⊕_		*Ac*, %
*Z*_1_	0.04 ± 0.003	0.03 ± 0.002	74	0.045 ± 0.003	0.033 ± 0.002	78	0.025 ± 0.002	0.028 ± 0.002	60
*Z*_2_	0.06 ± 0.004	0.045 ± 0.003	76	0.055 ± 0.004	0.04 ± 0.003	80	0.021 ± 0.001	0.031 ± 0.002	66
*Z*_3_	0.31 ± 0.017*	0.48 ± 0.029*	82*	0.43 ± 0.027*	0.87 ± 0.052*	88*	0.32 ± 0.018*	0.51 ± 0.029*	84*
*Z*_4_	0.44 ± 0.027*	0.69 ± 0.041*	86*	0.62 ± 0.039*	0.96 ± 0.062*	86*	0.38 ± 0.022*	0.62 ± 0.036*	88*

**Table 4:** Statistical moments of the first to fourth orders characterizing distributions (*LD*_0, 90_; *LD*_45; 135_; *CD*_⊗, ⊕_) of vagina wall histological sections of the patients from group 1 and group 2 and balanced accuracy of the method

*Z*_*i*_	*LD*_0, 90_		*Ac*, %	*LD*_45; 135_		*Ac*, %	*CD*_⊗, ⊕_		*Ac*, %
*Z*_1_	0.08 ± 0.005	0.065 ± 0.004	72	0.07 ± 0.004	0.05 ± 0.003	82	0.065 ± 0.004	0.046 ± 0.003	78
*Z*_2_	0.09 ± 0.006	0.07 ± 0.004	78	0.09 ± 0.006	0.06 ± 0.004	86	0.075 ± 0.005	0.057 ± 0.004	76
*Z*_3_	0.48 ± 0.028*	0.92 ± 0.0049*	90*	0.41 ± 0.025*	0.24 ± 0.014*	94*	0.29 ± 0.017*	0.12 ± 0.007*	92*
*Z*_4_	0.34 ± 0.019*	0.76 ± 0.042*	94*	0.32 ± 0.019*	0.18 ± 0.011*	92*	0.36 ± 0.021*	0.15 ± 0.008*	90*

**Table 5:** Balanced accuracy of polarization mapping methods for the polycrystalline structure of partially depolarizing tissue layers of a healthy and pathologically altered vagina wall

*Z*_*i*_	{*α*; *β*}	{M_ik_}	(*LB*_0, 90_; *LB*_45; 135_; *CB*_⊗, ⊕_)	(*LD*_0, 90_; *LD*_45; 135_; *CD*_⊗, ⊕_)
*Z*_1_	54–60%	58–62%	60–78%	72–82%
*Z*_2_	55–62%	60–64%	66–80%	76–86%
*Z*_3_	63–65%	65–69%	82–88%*	90–94%*
*Z*_4_	61–67%	64–68%	86–88%*	90–94%*

### Differential matrices of the first order of spatially structured fibrillar networks

Figures 2 and 3 present the maps and histograms of the distributions of the polarization-reconstructed distributions of the phase parameters (see Fig. 2) and the amplitude (see Fig. 3) anisotropy of the partially depolarizing layer of brain tissue.

The analysis of the obtained results revealed:

- A good correlation between the symmetry of the experimentally determined differential matrix of the first order and the theoretical data [37–43]:

$$ {\displaystyle \begin{array}{l}\left\langle \left\{{m}_{11}\right\}\right\rangle =\left\langle \left\{{m}_{11}\right\}\right\rangle =\left\langle \left\{{m}_{11}\right\}\right\rangle =\left\langle \left\{{m}_{11}\right\}\right\rangle; \\ {}\left(\left\langle \left\{{m}_{12}\right\}\right\rangle =\left\langle \left\{{m}_{21}\right\}\right\rangle \right);\left(\left\langle \left\{{m}_{13}\right\}\right\rangle =\left\langle \left\{{m}_{31}\right\}\right\rangle \right);\left(\left\langle \left\{{m}_{14}\right\}\right\rangle =\left\langle \left\{{m}_{41}\right\}\right\rangle \right);\\ {}\left(\left\langle \left\{{m}_{23}\right\}\right\rangle =-\left\langle \left\{{m}_{32}\right\}\right\rangle \right);\left(\left\langle \left\{{m}_{24}\right\}\right\rangle =-\left\langle \left\{{m}_{42}\right\}\right\rangle \right);\left(\left\langle \left\{{m}_{34}\right\}\right\rangle =-\left\langle \left\{{m}_{43}\right\}\right\rangle \right).\end{array}}. $$

- The asymmetric structure of the histograms of distributions of the linear and circular dichroism (*LD*_0; 90_; *LD*_45; 135_; *CD*_⊗; ⊕_) parameters (fragments (4)–(6) and, conversely, sufficiently symmetrical bell-shaped structure of histograms of distributions of the linear and circular birefringence (*LB*_0; 90_; *LB*_45; 135_; *CB*_⊗; ⊕_) parameters (fragments (1)–(3)). From the physical point of view, these facts can be related to the different multiplicities of the “non-absorbing” (phase anisotropy—“q-acts”) and “absorbing” (amplitude anisotropy—“k-acts”) interaction of laser radiation with optically anisotropic structures of biological tissue of brain—*q* ≻ ≻ *k*. This difference is also determined by the fact that in the red «section of the spectrum, the probability of absorption is significantly lower than that of Fresnel transformations of laser waves by birefringent refractive collagen fibrils formed by optically active protein molecules. Due to the influence of these two factors in accordance with the central boundary theorem [44], the average values of the linear (*LB*_0; 90_; *LB*_45; 135_) and circular (*CB*_⊗; ⊕_) birefringence appear to be almost normally distributed. On the contrary, the values of the linear (*LD*_0; 90_; *LD*_45; 135_) and circular (*CD*_⊗; ⊕_) dichroism parameters of collagen networks are distributed asymmetrically.
- The predominance of the mechanisms of linear birefringence and dichroism (average values and range of histogram variation presented in fragments (4), (5)) over optical activity and circular dichroism (fragment (6))—$$ {\displaystyle \begin{array}{l}L{B}_{0,90};L{B}_{45;135}>C{B}_{\otimes, \oplus };\\ {}L{D}_{0,90};L{D}_{45;135}>C{D}_{\otimes, \oplus}\end{array}} $$is due to the “developed” fibrillar structure of brain tissue.

The results of the statistical analysis of the distribution of the parameters of the optical anisotropy of the polycrystalline structure of the histological section of brain tissue are presented in Table 1.

The analysis of the data provided by the statistical analysis showed that due to different probability of absorption acts and phase shift formation between the orthogonal components of the laser radiation amplitude in the volume of brain tissue, significant differences (within one order of magnitude—highlighted in gray) are formed between the statistical moments of the 3st; 4th orders that characterize the distribution of the values of parameters *LB*_0, 90_; *CB*_⊗, ⊕_and*LD*_0, 90_; *CD*_⊗, ⊕_.

The predominance of linear birefringence and dichroism is quantitatively “detected” by large (1.5–2.5 times) values of average (*Z*_1_) and dispersion (*Z*_2_) of the distributions$$ {\displaystyle \begin{array}{l}L{B}_{0,90};L{B}_{45;135};\\ {}L{D}_{0,90};L{D}_{45;135}\end{array}} $$in comparison with distributions$$ {\displaystyle \begin{array}{l}C{B}_{\otimes, \oplus };\\ {}C{D}_{\otimes, \oplus}\end{array}} $$.

### Differential matrices of the first order of “islet” polycrystalline structures of parenchymatous tissue

Figures 4 and 5 present maps and histograms of the partially depolarizing layer of the rectum wall distribution$$ {\displaystyle \begin{array}{l}L{B}_{0,90};L{B}_{45;135};C{B}_{\otimes, \oplus };\\ {}L{D}_{0,90};L{D}_{45;135};C{D}_{\otimes, \oplus}\end{array}} $$.

The comparative analysis of the obtained data on the structure of the first-order differential matrices of partially depolarizing layers of rectal wall (see Figs. 4 and 5) and brain (see Figs. 2 and 3) tissues revealed statistically similar manifestations of the set of optical anisotropy mechanisms of the fibrillar network and of the ensemble of spatially non-structured “islet” protein chains. In particular, distributions of the linear and circular dichroism parameters are also asymmetric (see Fig. 5, fragments (4)–(6)), while those of the parameters of linear and circular birefringence are “bell-shaped” (see Fig. 4, fragments (4)–(6)). The revealed fact can be related to the large multiplicity of light scattering by phase-shifting biological nets and “islets”. Due to this (repeated averaging), the spatial-geometric structure of tissue samples of both types is largely graded.

An important distinctive feature of the polycrystalline structure of the rectum wall tissue is the commensurability of the mechanisms of the linear (fragments (4), (5)) and circular (fragments (6)) birefringence. The revealed fact is quantitatively “detected” by the close by magnitude values of the mean (*Z*_1_) and variance (*Z*_2_), which characterize the distributions $$ {\displaystyle \begin{array}{l}L{B}_{0,90};L{B}_{45;135};\\ {}L{D}_{0,90};L{D}_{45;135}\end{array}} $$and$$ {\displaystyle \begin{array}{l}C{B}_{\otimes, \oplus };\\ {}C{D}_{\otimes, \oplus}\end{array}} $$, Table 2.

Comparative analysis of the data presented in Tables 1 and 2 revealed the most sensitive diagnostic parameters—skewness $$ {Z}_3\left(\begin{array}{l}L{B}_{0,90};L{B}_{45;135};C{B}_{\otimes, \oplus };\\ {}L{D}_{0,90};L{D}_{45;135};C{D}_{\otimes, \oplus}\end{array}\right) $$ and kurtosis $$ {Z}_4\left(\begin{array}{l}L{B}_{0,90};L{B}_{45;135};C{B}_{\otimes, \oplus };\\ {}L{D}_{0,90};L{D}_{45;135};C{D}_{\otimes, \oplus}\end{array}\right) $$. The differences between the statistical moments of higher orders for distributions of differential matrix elements for the samples of spatially structured and parenchymatous tissues reach two to three times.

The obtained results were put in the basis of clinical application—differential diagnosis of pathological changes in the polycrystalline structure of partially depolarizing layers of biological tissues of human organs on the example of prolapse of genitals.

### Differential diagnosis of pathological changes in the polycrystalline structure of partially depolarizing layers of biological tissues

This part of the paper presents the results of a possible clinical application of the differential Mueller matrix mapping method for partially depolarizing layers of the vaginal wall of different physiological states (group 1 and group 2). The topicality of such studies is also due to the fact that this tissue is characterized by a high level of blood filling. Therefore, its histological sections contain a significant amount of light-scattering uniform elements of blood, as well as coagulated protein fibrillar structures. As a result, it is practically impossible to obtain optically thin (*τ* < 0.1), non-depolarizing sections of such tissue.

Maps and histograms of the distribution of the parameters of phase*LB*_0, 90_; *LB*_45; 135_; *CB*_⊗, ⊕_ (see Fig. 6) and the amplitude*LD*_0, 90_; *LD*_45; 135_; *CD*_⊗, ⊕_ (see Fig. 7) of anisotropy of the vaginal wall samples obtained for group 1 and group 2.

The comparative analysis of the histograms of parameter distributions of the mechanisms of phase (see Fig. 6) and amplitude (see Fig. 7) anisotropy of layers of the tissue of the vaginal wall of both groups confirmed the conclusion concerning the decrease of linear birefringence (*LB*_0; 90_; *LB*_45; 135_) and dichroism (*LD*_0; 90_; *LD*_45; 135_) levels in the case of prolapse of genitals. This is indicated by the decrease in the range of changes and value of the main extremes of the corresponding histograms (see Figs. 6 and 7, fragments (2b) and (4b)).

This can be explained by degenerative-dystrophic changes in the network of myosin fibrils of the spiral and longitudinal layers of the vaginal wall of patients from group 2. As a result of such changes (disorientation of the packing directions, thinning [32, 33]), the linear birefringence and dichroism decrease.

The level of optical activity (*CB*_⊗; ⊕_) and circular dichroism (*CD*_⊗; ⊕_) of samples of both types varies significantly. This is indicated by the similar structure of the extreme and the ranges of changes in the structure of the histograms*H*(*CB*_⊗; ⊕_) and *H*(*CD*_⊗; ⊕_) (see Figs. 6 and 7, fragments (6a) and (6b)). This can be explained by the fact that the concentration of circularly birefringent structures of the epithelial base of samples of a healthy and pathologically altered vaginal wall varies insignificantly. In order to determine the criteria for differential diagnosis of tissue samples of the vaginal wall, we used a well-tested method of statistical and information analysis, which is described in details in references [45, 46]. Here, we give a brief description of its main stages:

- in the operatively extracted (prolapse of the genitalia) wall of the vagina it were visually determined areas of healthy and pathologically altered tissue;
- two groups of samples were formed: histological sections of tissue from such areas;
- by means of histological method (“gold standard”) for each sample, its state was determined—“norm” (group1, 32 samples) or “pathology” (group 2, 32 samples);
- within the limits of each group of samples with the verified diagnosis, the average values (${\overline{Z}}_{i=1;2;3;4}$) and standard deviations (±2*σ*) of the magnitude of the statistical moments that characterize the distributions$$ \left(\begin{array}{l}L{B}_{0,90};L{B}_{45;135};C{B}_{\otimes, \oplus };\\ {}L{D}_{0,90};L{D}_{45;135};C{D}_{\otimes, \oplus}\end{array}\right) $$ were calculated;
- the parameter *Z*_*i*_ was considered statistically reliable if its average value ${{\overline{Z}}^{(1)}}_i$ in group 1 does not coincide with the value ${{\overline{Z}}^{(2)}}_i\pm 2\sigma$ in group 2 and vice versa;
- on this basis, the parameters ${Z}_{i=1;2;3;4}^{\ast }$of objective differentiation of the samples of both groups were determined.

For possible clinical application of the method of differential Mueller matrix mapping for each group of samples traditional for evidence-based medicine [42, 43], operating characteristics that define the diagnostic potentiality of the method were determined. Namely—sensitivity ($Se=\frac{a}{a+b}100\%$); specificity ($Sp=\frac{c}{c+d}100\%$); balanced accuracy ($Ac=\frac{Se+ Sp}{2}$), where a and b are the numbers of correct and wrong diagnoses within group 2; c and d are the same within group 1.

Tables 3 and 4 show the values (${\overline{Z}}_{i=1;2;3;4}\pm 2\sigma$) of the statistical parameters that characterize the$$ \left(\begin{array}{l}L{B}_{0,90};L{B}_{45;135};C{B}_{\otimes, \oplus };\\ {}L{D}_{0,90};L{D}_{45;135};C{D}_{\otimes, \oplus}\end{array}\right) $$distributions of vagina wall histological sections of the patients from group 1 and group 2.

The comparative analysis of the set of statistical moments of the first to fourth orders revealed the following ones, the most sensitive to changes in phase and amplitude anisotropy of vagina wall layers (highlighted in gray). As in the case of analysis of the polycrystalline structure of fibrillar (see Table 1) and parenchymal (see Table 2) tissues, the most diagnostically efficient parameters were the statistical moments of the third and fourth orders:

- *Z*_3; 4_(*LB*_0, 90_; *LB*_45; 135_; *CB*_⊗, ⊕_)—good level *Ac* > 80% (see Table 3);
- *Z*_3; 4_(*LD*_0, 90_; *LD*_45; 135_; *CD*_⊗, ⊕_)—excellent quality *Ac* > 90% (see Table 4).

This is arguably related to a high probability of scattering of laser radiation by optically anisotropic structures of the vagina wall tissue. Due to this, the distribution histograms of phase anisotropy *H*(*LB*_0, 90_; *LB*_45; 135_; *CB*_⊗, ⊕_) are close to the “bell-like” ones (see Fig. 6). Therefore, the difference between asymmetry and excess (*Z*_3; 4_(*LB*_0, 90_; *LB*_45; 135_; *CB*_⊗, ⊕_)) of such distributions are less pronounced than in the case of similar statistical parameters *Z*_3; 4_(*LD*_0, 90_; *LD*_45; 135_; *CD*_⊗, ⊕_), which characterize the distribution of the amplitude anisotropy.

### Comparative studies of the diagnostic efficiency of direct polarization, Mueller matrix, and differential matrix mapping

In this part of the paper, we present comparative information on the balanced accuracy of our method and other most commonly spread polarimetric techniques—direct polarization (coordinate distributions of the azimuth *α* and polarization ellipticity *β*) [27] and Mueller matrix (MMI{M_ik_}) [28, 29] mapping of partially depolarizing biological tissues (see Table 5).

Comparative analysis of balanced accuracy of diagnostic methods of the change in phase and amplitude anisotropy of partially depolarizing layers of vagina wall tissue showed

- Polarization mapping {*α*, *β*} is not applicable (Ac < 70%) for differential diagnosis of the vagina wall with genital prolapse.
- Accuracy of differential diagnosis by direct Mueller matrix mapping {M_ik_} method reaches satisfactory (Ac = 71–72%) level.
- Efficiency of differential matrix mapping lies within good (Ac > 80%) and excellent quality (Ac > 90%) (Table 5) (highlighted in gray).

## Summary and conclusion

1. The differential Mueller matrix mapping method for reconstruction of distributions of linear and circular birefringence and dichroism parameters of partially depolarizing layers of biological tissues of different morphological structures is introduced and practically implemented.
2. Coordinate distributions of values of the parameters of phase and amplitude anisotropy of histological sections of brain tissue with spatially structured optically anisotropic fibrillar network, as well as of parenchymatous tissue of rectum wall with the “islet” polycrystalline structure, were determined.
3. Within the statistical analysis of polarizationally reconstructed distributions of the averaged parameters of phase and amplitude anisotropy, significant sensitivity of the statistical moments of the third and fourth orders to the changes of the polycrystalline structure of partially depolarizing layers of biological tissues was determined.
4. Differential diagnostics of changes in the phase (good balanced accuracy) and amplitude (excellent balanced accuracy) of the anisotropy of the partially depolarizing layers of the vagina wall tissue with prolapse of the genitals is realized.
5. The maximum diagnostic efficiency of the first-order differential matrix method was demonstrated in comparison with the traditional methods of polarization and Mueller matrix mapping of histological sections of light-scattering biological tissues.
